# Social and Psychophysiological Consequences of the COVID-19 Pandemic: An Extensive Literature Review

**DOI:** 10.3389/fpsyg.2020.580225

**Published:** 2020-12-16

**Authors:** Vicente Javier Clemente-Suárez, Athanasios A. Dalamitros, Ana Isabel Beltran-Velasco, Juan Mielgo-Ayuso, Jose Francisco Tornero-Aguilera

**Affiliations:** ^1^Faculty of Sports Sciences, Universidad Europea de Madrid, Madrid, Spain; ^2^Grupo de Investigación en Cultura, Educación y Sociedad, Universidad de la Costa, Barranquilla, Colombia; ^3^Studies Centre in Applied Combat, Toledo, Spain; ^4^Laboratory of Evaluation of Human Biological Performance, School of Physical Education and Sport Sciences, Aristotle University of Thessaloniki, Thessaloniki, Greece; ^5^Departamento de Educación, Universidad Antonio de Nebrija, Madrid, Spain; ^6^Department of Biochemistry, Molecular Biology and Physiology, Faculty of Health Sciences, University of Valladolid, Soria, Spain

**Keywords:** COVID-19, pandemic, stress, anxiety, depression, nutrition, gut, physical activity

## Abstract

The Coronavirus Disease 2019 (COVID-19) pandemic, now a global health crisis, has surprised health authorities around the world. Recent studies suggest that the measures taken to curb the spread of the COVID-19 outbreak have generated issues throughout the population. Thus, it is necessary to establish and identify the possible risk factors related to the psychosocial and psychophysiological strain during the COVID-19 outbreak. The present extensive literature review assesses the social, psychological, and physiological consequences of COVID-19, reviewing the impact of quarantine measures, isolation, vast human loss, social and financial consequences in the family’s economies, and its impact on the psychological health of the population. We also discussed the effect of psychophysiological factors, considering the impact of physical inactivity and modifications in nutritional habits, at psychological and physiological levels. The present review includes an actualized to date bibliography, articles for which were methodologically analyzed to verify they met the standards of quality and scientific accuracy. Authors understand the pandemic as a multifactorial event for which only a profound and extensive analysis would lead to better compression and efficient intervention in the near future.

## Introduction

Since the emergence of severe acute respiratory syndrome (SARS)-Cov-2 in the city of Wuhan, (Hubei, China) in December 2019, governments around the world have taken unprecedented actions to respond and contain the virus (Vital Surveillances, 2020). Countries are implementing different community, economic, and public health control measures to flatten the epidemic curve and avoid the overload and possible collapse of their health systems. The quick response by governments is strictly necessary since epidemiologists predict 7.0 billion infections and 40 million deaths globally for the year 2020 if no interventions are done (Walker et al., 2020). To date (22th June of 2020), the ongoing SARS-Cov-2 pandemic has resulted in 9,053,536 active infected cases, with a total of 4,482,312 recovered patients and 470,844 deaths (WHO Coronavirus Disease (COVID-19) Dashboard, 2020). These large and rapid increases in the confirmed cases and deaths are causing medical personnel and the general population to experience psychological health problems, including anxiety, depression, and stress (Kang et al., 2020). Studies suggest that frontline nurses present lower vicarious traumatization scores than non-frontline nurses and the general public, possibly due to their reduced accessibility to formal psychological support, less first-hand medical information on the outbreak, or less intensive training on personal protective equipment and infection control. However, a survey of 500 health care workers showed that 14.5% screened positive for anxiety, 8.9% for depression, 6.6% for stress, and 7.7% for clinical posttraumatic stress disorder (PTSD; Tan et al., 2020).

In order to attenuate the reproduction/infection cases, suppression measures are being taken worldwide to bring the *R* number below 1 with the use of non-pharmaceutical interventions. These measures must be taken till a vaccine is available, which according to recent data is likely at least 12–18 months away (James et al., 2020). These types of actions focus on the restriction of free movement and assembly, quarantining people with absolute diligence. According to the World Health Organization (WHO), these suppression strategies will allow the scientific community and governments to have time until a vaccine or an effective treatment becomes widely available. However, quarantine is an unpleasant experience for those who undergo it. Separation from loved ones, the loss of freedom, and uncertainty over disease status creates dramatic effects. Although the majority of people are not expected to suffer mental disorders in the actual pandemic, a significant percentage of the population will still experience intense emotional adjustment reactions (Li et al., 2020). The impact of prolonged confinement, in addition to the death of relatives and increased social adversity, may lead to psychological adverse effects, increasing the risk of emotional disturbance, depression, low mood, irritability, insomnia, and post-traumatic stress symptoms (Duan et al., 2020). Authors relate how loss of appetite, mood swings, delusions, fear, sleep disorders, and suicidal/domestic-violence cases have become quite common during lockdowns with helpline numbers being overload with people seeking help (Ammar et al., 2020b). Since authors have stated the high risk of psychosocial strain during the current home confinement period and have provided a clear remit for the urgent implementation of a technology-based intervention to foster an Active and Healthy Confinement Lifestyle (AHCL; Ammar et al., 2020a, b), an accurate understanding of behavioral changes accompanying the Coronavirus Disease 2019 (COVID-19) lockdowns is a necessary step. Quarantine and confinement measures have been taken with the main objective of alleviating the medical systems, which were in danger of collapse, but the remedy cannot be worse than the disease. However, psychological care services can be highly affected, collapsing as a consequence of the measures for health prevention. Regardless of whether mobility restriction measures are being progressively lifted in most countries, we must be prepared for a rebound in the disease, or a new wave in the appearance of the virus, which clearly indicates the need to produce guidance for the public.

Thus, the aim of the present review was to examine the present literature and provide a comprehensive narrative explanation of the psychophysiological and social effects of the COVID-19 pandemic. Since a second wave of the virus is likely to appear, it is essential to know the consequences of COVID-19 on the human psyche and physiology.

## Psychosocial and Psychological Stressors in the Pandemic

Numerous studies, mostly using questionnaires, have been developed to identify how confinement has impacted the health of the world population, aiming at identifying possible relationships between psychosocial, psychological, and behavioral changes during the confinement period and obtaining scientific data that could help to characterize the psychosocial aspects of the COVID-19 crisis (Ammar et al., 2020a, b). In general, authors describe how the COVID-19 pandemic has impacted the mental health of citizens worldwide; isolation measures, fear, uncertainty, and economic turmoil, lack of social connectedness and trust in other people and institutions, and jobs and incomes being at risk are taking a huge toll in terms of anxiety and worry, leading to “psychological distress” (Zhang J.J. et al., 2020). The United Nations (UN) has warned of the necessity for a policy briefing, calling for substantial investment in support services (Howard, 2020). Depression and anxiety cost the global economy more than one trillion dollars per year, and this was before the COVID-19 pandemic, and globally the ratio of mental health professionals per citizen is 1 to 10,000 (Chisholm et al., 2016). Although anxiety and depression affects over 264 million citizens worldwide, only 2% of the healthcare budget on average goes on mental health services (Sobocki et al., 2006).

Due to the novelty of the virus, there is little evidence available on how this new global political, economic, and health scenario may impair the psychological health of subjects. Researchers have suggested that new psychiatric symptoms in people without mental illness would increase, and that people with pre-existing mental illness would aggravate their symptomatology (Ho et al., 2020). Independently of the exposure to the virus, the experience of depression and anxiety and fear of falling sick or dying may potentially trigger mental breakdowns (Hall et al., 2008). Previous research has revealed a large spectrum of psychological impacts that outbreaks could inflict on people. Increasing psychiatric morbidities vary from depression, anxiety, panic attacks, somatic symptoms, and PTSD symptoms, to delirium, psychosis, and even suicidality (Yang et al., 2020). However, effects may vary according to the psychological trigger.

### Imminent Psychological Effects, the Fear, and the Normalization Process

The health alert state declared by the WHO due to the COVID-19 pandemic merited this the greatest challenge faced by societies since the Influenza virus. Countries like the United States, France, the United Kingdom, Spain, or Brazil were forced to adopt policies without precedents to prevent the spread of the virus (Thakur and Jain, 2020). These actions were mostly taken only when fear was already evident in society and involved the restrictions of free movement, closure of airports and shops, and the total confinement of the population (Xiao et al., 2020a). Thus, within the space of 1 day, citizens went from experiencing a generalized feeling of uncertainty and lack of information to being notified of their confinement in their homes to avoid infection and the collapse of the health systems. Countries found themselves unable to cope with the increasing number of daily cases and to administer correct treatments due to the lack of technical and human resources (Liu et al., 2020). Fear was evident in society, from fear of contagion, fear of how the virus was transmitted, fear of the future, of losing jobs, to fear for loved one’s health (Shanafelt et al., 2020).

Therefore, citizens are experiencing an extremely stressful and novel living situation, one which is impacting each subject in different ways and undoubtedly leading to psychological consequences in the medium and long term (Holmes et al., 2020). Confinement is an anomalous situation that forces people to modify their normal life habits and daily routine (Girdhar et al., 2020). Suddenly, normal activity comes to a halt; even in cases where it is possible to continue with work, the person modifies their natural way of acting: they no longer wake up every day to the rhythm of the alarm clock, do not get dressed and clean, do not leave the house to go to work, do not take public or private transport to get to their work location, do not interact for at least 8 h a day with their colleagues, and they do not organize the rest of the day to attend to other family and social obligations (Venkatesh and Edirappuli, 2020).

During confinement, the person is forced to suddenly stop their life, and although in the first days a brief relief is felt due to this stop, like that produced during the vacation period, the person quickly begins to present symptoms associated with anxiety produced by social isolation, lack of mental hygiene habits, and repeated exposure to negative news and information. Thus, feelings of sadness, apathy, fear, uncertainty, frustration, lack of impulse control, anxiety, alterations of the circadian cycles, insomnia, hypervigilance, or difficulties in concentrating may appear (Kumar and Nayar, 2020). In this line, it is important to consider that people with mental pathologies existing before the pandemic should be extremely cautious about the fulfillment of some rules that prevent a worsening of the symptoms associated with their diagnosis (Tsamakis et al., 2020). While for people without previous pathologies, it should be equally important to be aware to apply different prevention actions to maintain a balanced mental health during the complex and unusual situation of social isolation in the pandemic (Ahmed et al., 2020).

Consequently, it would be indispensable for people to create a series of habits that protect their mind from these associated negative factors (Pancani et al., 2020). Amongst these, previous authors highlighted the need to control exposure to the news, and to limit choices to the most reliable sources. People are encouraged to try to normalize the day-to-day by creating healthy routines, maintaining as far as possible those that we had previously internalized, such as exercising at a certain time each day. Daily rituals should be established if possible; when the subject can maintain his/her work activity, it was recommended to maintain a more or less established schedule, trying to separate personal life from work. When a person cannot maintain such activity, a reasonable part of the day should be dedicated to actively seeking employment, then the feeling of having been productive can increase. Personal care rituals should also be practiced as we would do daily (Lippi et al., 2020).

In short, in a state as exceptional as the one society is experiencing now, we must act in the most rational way possible to try to prevent not only the worsening of the pandemic but also the exacerbation of mental disorders.

### From Social Beings to Total Isolation: The Imposed Quarantine

The current situation produces a new social feeling not previously experienced by society, wherein there may be certain level of distrust toward other individuals in terms of disease spread and toward the government and healthcare services in terms of their capability to contain the outbreak (Ho et al., 2020). In previous pandemics, such as the SARS, confinement resulted in symptoms of PTSD and depression in Canadians (28.9% and 31.2% of the population, respectively; Lesser and Nienhuis, 2020). Specifically, hospital staff who quarantined for 9 days developed acute stress disorder, reporting exhaustion, detachment from others, anxiety when dealing with febrile patients, irritability, insomnia, poor concentration, indecisiveness, deteriorating work performance, and reluctance to work or consideration of resignation (Bai et al., 2004). In this line, parents and children who were in quarantine presented with four times higher stress scores than ones who were not in quarantine (Sprang and Silman, 2013).

In the case of the COVID-19 epidemic in China, researchers found a prevalence of 45.3% for moderate and severe depressive symptoms, of which 84.7% spent between 20–24 h a day confined at home (Wang et al., 2020). Recent research suggests that there is an emotional impairment caused by extreme fear and uncertainty (Shigemura et al., 2020). Furthermore, and given the uncontrolled fear and distorted risk perception, symptomatology of insomnia, anger, extreme fear of the disease, fear to leave the house, increased use of drugs and tobacco, and social isolation were reported (Wang et al., 2020). Authors found that home confinement due to COVID-19 evoked a negative effect on mental well-being and emotional state and these psychosocial rates were associated with unhealthy lifestyle behaviors, with a greater proportion of individuals experiencing physical and social inactivity, poor quality sleep, unhealthy eating habits, and unemployment (Ammar et al., 2020b). An analysis of the Chinese social media Weibo found that negative emotions, such as outrage, depression, and anxiety, significantly increased after the declaration of the COVID-19 pandemic, leading to a decrease in life satisfaction (Li et al., 2020). This information is in line with further studies in the Chinese population, where nearly 20% reported moderate to severe depressive symptoms, and almost 30% reported symptoms of moderate to severe anxiety, with over 85% of the participants spending around 20 h confined at home a day (Wang et al., 2020). In this line, previous studies suggested that larger hours of isolation and quarantine are associated with a larger prevalence of PTSD and depressive symptoms, establishing a direct correlation with time (Hawryluck et al., 2004).

However, once again, the experience of anxiety symptoms and psychological disorders may be modulated through several triggers, which can aggravate the quarantine situation, its perception, and somatization (Brooks et al., 2020). One of the most important elements is the duration of the quarantine since authors relate a directly proportionate relationship with psychological disorders, showing greater post-traumatic stress symptoms, avoidance behaviors, and anger during longer periods (Reynolds et al., 2008). In addition, the creation of a vicious circle of routine can lead to frustration and boredom, as well as the loss of a routine and the high sense of isolation from the rest of the world by the imposed law (Wilken et al., 2017). A lack of information is a further aggravator of symptoms, with surveys exposing how participants cited poor information from health authorities as a powerful stressor, making the uncertainty of the context greater, thereby increasing fear (Di Giovanni et al., 2004; Cava et al., 2005; Caleo et al., 2018).

Undoubtedly, quarantine and isolation are perceived as a negative and stressful stimulus, leading to psychological discomfort. Interestingly, the psychological scar of quarantine can be found months and even years after the quarantine happened, suggesting acute and chronic effects (Jeong et al., 2016; Liu et al., 2020). Thus, along with the quarantine measures imposed by governments, psychological assistance, planning, and measures must be implemented, not only during the quarantine period but also in the subsequent de-escalation.

### Experiencing COVID-19 Symptomatology

Experiencing sickness may trigger shame, social stigma, and even the feeling of guilt. A large survey of 730 COVID-19 positive patients from China showed a 96.2% prevalence of post-traumatic stress symptoms associated with the COVID-19, of which 49.8% considered psycho-educational services helpful (Bo et al., 2020). When compared to previous outbreaks like the 2003 SARS, the prevalence of PTSD in SARS survivors was 9.79% in their early recovery phase (Fang et al., 2004) and 25.6% at 30-month post-SARS (Mak et al., 2020). In the actual pandemic, the authors concluded that patients who experienced COVID-19 infection have a 29.2% increased prevalence of depression. Also, trends for an increased prevalence of depression comorbid with anxiety were identified in patients who experienced COVID-19 infection (21.1%) and the general population (22.4%; Zhang J. et al., 2020).

Among COVID-19 patients, there are certain groups which are likely more exposed to and suffer greater psychological impact from than the quarantined population, such as health workers. Authors reported in this collective a 14.5% prevalence of anxiety, 8.9% of depression, 6.6% of stress, and 7.7% of PTSD (Tan et al., 2020). Cross-sectional studies of self-rated questionnaires in medical staff treating COVID-19 patients showed how greater levels of anxiety scores positively correlate with stress and negatively with sleep quality, social support, and self-efficiency (Xiao et al., 2020b). Interestingly, traumatization related to COVID-19 was higher among non-front-line than front-line nurses, while traumatization was lower among the general public and higher for front-line nurses but not non-front-line nurses (Li et al., 2020). Finally, the presence of any COVID-19 symptoms and the self-perceived poor health state were largely associated with higher rates of anxiety, depression, and the possible need for psychological assistance (Rajkumar, 2020).

### Vast Human Loss

Since the tendency of deaths continues to rise, knowledge of the idiosyncratic nature of loss and grieving its essential. The grieving process is a completely normal and expected reaction which reflects a unique convergence of responses, from affective, behavioral, physical, and cognitive ones. Psychologists pointed out that the death of a loved one should not be categorized as a mental illness but, rather, as a process of recovery from an imbalance, a return to homeostasis. However, due to the actual situation and the inability to attend the last hours of life and to provide a decent burial, this has led to the impossibility of gaining closure, leading to an ambiguous loss, thus stimulating feelings of resentment (Shear et al., 2020).

Also, patients, family members, and loved ones under the COVID-19 symptomatology may experience the possibility of anticipatory grief. For health care workers, the discontinuation of palliative care or disconnection from mechanical ventilation systems is perceived as emotionally onerous and psychologically difficult, but little time is allowed for mourning due to the intense situation. For families, they know that they will miss the final moments in case of death, will be unable to say goodbye, and know that their loved ones are suffering without being able to assist them. Therefore, as a result of prolonged and disabling grief, a large number of the population is at risk of prolonged grief disorder (PGD) in this pandemic, which will compromise mental wellbeing (Zhai and Du, 2020).

Specific strategies to help grievers to adapt to loss in these highly challenging times are essential. A general approach could be following the model of William Worden, which states that acceptance of the loss is the first step in healing (Worden et al., 2020). The appearance of mental and psychological blocks may be usual as a consequence of the shock due to the sudden disappearance with the impossibility of a goodbye. Two possible scenarios may be established, negation or indifference, which will indicate an unprepared duel. In these situations physical actions, spiritual connections, and rituals may be helpful for the patient to gradually accept the irreversibility of the situation and the human loss. In this line, a home-based ritual, letters to the deceased, or reorganizations associated with objects are strategies that are easily achievable at home and may be of substantial helpful. Secondly, it is necessary to work on emotions and pain since the situation will lead to a collapse or the manifestation of depressive symptoms. Subsequently, an adaptation phase is reached, and the person accepts an environment in which the deceased is absent. If the previous phrases are correctly processed, the person should be able to emotionally relocate the deceased, beginning to resume their normal way of life while still remembering their loved one.

### Financial Collapse, the Fall Dawn of Personal Economies

Coronavirus Disease 2019 has led to a global economic recession (Fernandes, 2020), rating the Economic Cooperation and Development (OECD) losses between 2.3 and 4.4% of gross national product worldwide (Organisation for Economic Co-operation and Development, 2014). This is a direct consequence of the reduction in jobs, as well as a marked loss in productivity (Melnychuk, 2012), with labor markets playing a fundamental role in mental health results (Gili et al., 2013).

Worldwide, confinement policies have forced large corporations, financial markets, and small businesses to stop suddenly. The reality after this situation is having a catastrophic effect on economies and resulting in a vast loss of employment (Pfefferbaum and North, 2020; Piguillem and Shi, 2020). One of the largest world economies, the United States of America, suggests a loss of employment in the second quarter similar to that experienced during the great recession, with a 30% drop in Gross Domestic Product (GDP; Kennedy, 2020). For European Union countries such as Spain, one of the most affected by the pandemic, the amount of unemployed people registered a 14.5% increase during the first three weeks of the alarm state decreed by the government, making it the country in Europe with the second highest number of unemployed people (Woolhandler and Himmelstein, 2020).

Indeed, work activity is among the most important factors for the development of the individual, since it not only consolidates a way of obtaining economic income but is also an essential factor for personal and societal development (Monitor, 2020). The individual who exercises stable work activity will be able to develop both individually and collectively, and develop emotional ties with other individuals, generating relations of equality, respect, and collaboration that will form part of their personality and that will directly impact their self-image and self-concept (Fernandes, 2020). Therefore, employment is of great value for the person and is not only a functional fact; it has psychological and social implications since the individual can establish future projections and planning of work level and personal identity (Fleming et al., 2019). Some authors related that the effects on people when they lose their jobs are going to be just as devastating in social and health terms as the spread of the health pandemic (Douglas et al., 2020).

Currently, there is extensive scientific literature that links unemployment and the mental health of workers, establishing a strong inverse relationship (Paul and Moser, 2009). Feelings of depression, anxiety, psychological distress, and decreased life satisfaction are direct indicators of psychological illnesses and psychiatric morbidity (Boyle et al., 2020). When unemployment is prolonged, other symptoms may appear, such as hopelessness about the future, not being able to understand one’s own feelings, a decrease in self-esteem, and chronic stress, which may lead to a feeling of listlessness and negative helplessness (Pfefferbaum and North, 2020). The immediate changes in social stage, loss of self-concept and identity, decrease in social contacts, financial deprivation, and uncertainty about the future have been the main mechanisms that explain the appearance of psychological pathologies (Extremera and Rey, 2016). However, it must be considered that the possible psychophysiological impact of job loss would depend on family circumstances, for example by increasing financial stress (Draghi, 2014), generating economic instability in the family unit (Maitoza, 2019), influencing marital conflicts, and increasing the possibility of divorce (González-Val and Marcén, 2018). There is a clear tendency to consume alcohol and different drugs such as benzodiazepines, cannabis, or other addictive substances in an attempt to prevent these negative emotions from appearing. Addiction becomes a refuge in the face of a lack of stability and to try to silence the most emotional and negative parts of the loss of employment (Nurmela et al., 2018). It is important to note that longitudinal studies have shown that although life satisfaction is moderately stable over time, individuals strongly reacted to unemployment and did not completely return to their previous satisfaction and happiness levels, even after becoming employees (Lucas et al., 2004). In addition, according to Ammar et al. (2020b), the negative psycho-emotional effect of COVID-19 was also shown to be accompanied by a negative effect of unemployment compared to before the confinement period.

On a physiological level, unemployment could elicit alterations in sleep cycles, chronic fatigue, exhaustion, and physical strain that could occur, facilitating continued muscular pain (Antillón et al., 2014). All of these symptoms would be present due to the excess in cortisol production, which regulates these physiological responses to stress and is associated with a wide range of diseases and alterations in areas of the hippocampus. Memory loss and difficulties in the correct functioning of higher executive functions, such as attention and information processing, among others, may also be experienced (Musana et al., 2019). From a long term perspective, unemployment has been associated with increased cardiovascular diseases (Johansson et al., 2020), chronic inflammation (Sumner et al., 2020), abusive consumption of antidepressants, tobacco, and alcohol (Kaiser et al., 2017), decreased immune function (Sumner and Gallagher, 2017), and an 11% increase in all-cause mortality (Browning and Heinesen, 2012). In this line, the need for a specific approach to unemployed individuals that takes into account their vulnerability should be considered.

## Physiological Stressors in the Pandemic

Severe acute respiratory syndrome-CoV-2 has shown a high virulence that has caused a rapid increase in infected and deceased patients. Currently, without a developed vaccine and population herd immunity, it is an individual’s immune system that must face this virus (Clemente-Suárez et al., 2020). In this regard, different behavioral factors have a direct effect on the immune system and the organic state of the subject, also affecting their mental state (Anderson, 2020). Nutritional habits, physical activity, and even the individual’s microbiota are be factors that could play an important role in the fight against COVID-19.

### Physical Inactivity in the Pandemic

Social isolation measures imposed by governments across the world caused an abrupt decline in physical activity, as recreation facilities, athletic centers, gyms, public parks, playgrounds, and schools were forced to close. As it may take some time for communities to return to normal daily life, the longitudinal effects of physical inactivity are still not noticeable. Still, several studies conducted through online surveys measured the effects of social isolation on physical activity in various populations, given the fact that prolonged inactivity can lead to various health issues and potential remedies (Lippi et al., 2020).

There are recent studies that showed the impact of confinement on the physical activity habits of citizens. One of the first ones found insufficient physical activity in 60% of Chinese citizens during the early days of the pandemic (Qin et al., 2020). They also showed a negative effect of social isolation on physical activity during various intensities, from vigorous exercise to walking, while daily sitting time presented an increase from 5 to 8 h daily (Ammar et al., 2020a).

In Australia, biomedical students of both sexes were found to have reduced physical levels by 30%, compared to the pre-pandemic years (2018 and 2019), despite the participants’ report for sufficient activity levels (Gallo et al., 2020). Meanwhile, the% of United Kingdom citizens of both sexes (20 years old) reported equal levels of physical activity intensity during the lockdown period, compared to the pre- pandemic period. These findings were more obvious for males, while other factors, such as living alone or the existence of a garden in the house, also influenced the results (Rogers et al., 2020). In adult Canadians, 22.4% of the participants that were self-reported as “active” became less active, while 40.3% became more active. For those reported as “inactive,” 40.5% became less active and 33% became more active (Lesser and Nienhuis, 2020). In the Spanish population (age range 18- to 64-years-old) a decrease in vigorous physical activity and walking time (16.8% and 58.2%, respectively), with a simultaneous increase of sedentary time (23.8%), were reported. Males reduced the time for vigorous physical activity to a greater degree compared to females (21% vs. 9%, respectively). Finally, young participants (18- to 24-years-old) and students had the highest decrease in moderate activities and walking time (Castañeda-Babarro et al., 2020). Finally, using a microsimulation model, childhood obesity under four different scenarios related to school closure in the United States was analyzed, reporting an increase in the obesity rate of 2.4% during the ominous scenario (school closure until December 2020) with a modestly higher impact in specific populations (i.e., boys, non-Hispanic black children, and Hispanics; An, 2020; Chen et al., 2020; Hammami et al., 2020; Ricci et al., 2020).

It is obvious that the quantification of the impact and extent of COVID-19 on physical inactivity is largely dependent on the lockdown duration and the transition phase to “normal life” strategies imposed by different governments. Given the importance of staying physically active, useful home-based activities are already available through different studies (Chen et al., 2020; Hammami et al., 2020; Ricci et al., 2020) and the American College of Sports Medicine, 2020 (3/2020). In addition, authors developed a proposal of physical performance tests adapted as home workout options during the COVID-19 pandemic, with simple home-based exercises, considering individual limitations. According to these authors, individuals might monitor their performance daily and employ useful home-based exercise strategies to counterbalance the negative impact of the sedentary lifestyle during confinement. Therefore, they recommend the practice of physical exercise (easy, useful, and suitable) to be performed in a small physical space, such as at home without special devices (da Cunha de Sá-Caputo et al., 2020). Moreover, specific populations, such as cardiac patients, are also encouraged to participate in home-based activity programs, under online supervision, aiming to minimize premature mortality (Peçanha et al., 2020).

Being a disease primarily affecting the respiratory system (Yuki et al., 2020), greater attention has been given to moderate aerobic exercise as a proposed strategy to improve lung function, decrease hospitalization (Mohamed and Alawna, 2020), enhance the immune system (Zbinden-Foncea et al., 2020), prevent respiratory infections (Olivo et al., 2014) and, consequently, prevent new COVID-19 incidences (Dixit, 2020). Complementary, the role of low- to medium-intensity resistance training to prevent neuromuscular degeneration is highlighted (Narici et al., 2020).

### Nutrition in the Pandemic

The relationship between nutritional status and the prevention and treatment of COVID-19 means that several dietary-nutritional aspects can be worked on. In this sense, although there is no specific diet that can prevent COVID-19, a balanced diet and adequate hydration favors presenting a stronger immune system and having a lower risk of chronic and infectious diseases (Naja and Hamadeh, 2020). Therefore, it is recommended to eat a variety of fresh and unprocessed foods every day to obtain the vitamins, minerals, dietary fiber, proteins, and antioxidants that the body needs in order to prevent any infectious disease, including COVID-19 (Jayawardena et al., 2020). In addition, the consumption of sugar, fat, and salt that promote comorbidities (obesity, cardiovascular diseases, and diabetes, among others) that have been associated with having COVID-19 should be reduced (Zhou et al., 2020). Along with these dietetic recommendations, supplementation with 10,000 IU/day of vitamin D for a few weeks (≈4 weeks) and subsequent maintenance of 5,000 IU/day could be suggested due to the direct relationship that has been shown between low concentrations of 25-hydroxyvitamin D [25 (OH) D] and serious complications of COVID-19 (Grant et al., 2020). This may be because vitamin D is important in regulating the physical barrier, natural cellular immunity, and adaptive immunity (Rondanelli et al., 2018). However, although supplementation with probiotics has shown a direct relationship with the immune system and could prevent all types of infections (Kanauchi et al., 2018), the blind use of conventional probiotics for the treatment of COVID-19 is not recommended until understanding of SARS-CoV-2 pathogenesis and its effect on the intestinal microbiota is gained (Mak et al., 2020). These same recommendations could be applied to people who have emerged from a confined situation and in which physical inactivity together with an excess of energy has caused them to have a higher risk of suffering metabolic disorders (Martinez-Ferran et al., 2020).

Concerning the best nutritional strategy in the treatment of people infected with COVID-19, this will depend on the state of health of the patient. For those patients who are not in a critical situation, diets containing at least 25–30 kcal/day with foods of different textures and consistencies that are easily digestible (yogurt, custard, fruit mousse, fruit slices, and soft cheese, etc.) should be recommended (Caccialanza et al., 2020). This type of diet in addition to ensuring adequate energy content will ensure an optimal content of vitamins and minerals, as well as protein (Zhang and Liu, 2020). However, for those patients who have intake problems due to COVID-19 (Beltrán-Corbellini et al., 2020), supplementation with 20 g/day of whey protein may be considered together with a supplement that includes vitamins and minerals that cover daily recommendations (Caccialanza et al., 2020). Whey protein supplementation is based on its anabolic, antioxidant, and immunomodulatory (Cross and Gill, 2000) properties and its potential antiviral activity (Ng et al., 2015), combined with a high digestibility (Ha and Zemel, 2003). In addition, vitamin D supplementation [50,000 IU/week if 25 (OH) D < 20 ng/ml; 25,000 IU/week if 25 (OH) D ≥ 20 to < 30 ng/ml] would be justified (Caccialanza et al., 2020) in order to reduce levels of inflammation and immune activation, and increase immunity against pathogens (Grant et al., 2020).

On the other hand, although enteral nutrition in the ICU has been shown to be feasible and safe (Pan et al., 2020), its implementation in patients with COVID-19 could be difficult due to frequent gastrointestinal symptoms in these patients (vomiting and diarrhea; Saez de la Fuente et al., 2016). Furthermore, hypoxemia requires delayed enteral nutrition (Singer et al., 2019). However, in patients with acute respiratory distress syndrome/acute lung injury, enteral diets containing eicosatetraenoic acid, gamma-linolenic acid, and antioxidant agents may offer a clinical benefit in oxygenation and ventilation days (Singer et al., 2019).

Finally, in situations of quarantine and/or confinement, an appeal must be made to nutritional moderation since the decrease in physical activity is large. An excess of energy in a situation of physical inactivity can increase metabolic disorders that increase the risk of multiple chronic diseases (Winn et al., 2019). Consequently, the recommendation during a period of confinement is to eat a balanced and healthy diet with a restricted calorie intake, avoiding an over-intake. In this line, using the SDBQ-L, a short, crisis-driven questionnaire recently developed to assess eating behaviors before and during the blocking period, Ammar et al., 2020a, reported that food consumption and meal patterns (the type of food, eating out of control, snacks between meals, number of main meals) were more harmful to health during confinement, with only excessive alcohol consumption decreasing significantly. In their study, in responses to the diet behavior questionnaire before and during home confinement, they found that consuming unhealthy food, eating out of control, the number of snacks between meals or late-night snacking, and the numbers of main meals were significantly higher during home confinement. Thus, the diet should be based on low glycemic carbohydrates (vegetables, legumes, or fruits), foods rich in healthy fats (olive oil, salmon, or nuts), and foods rich in protein with low-fat content (skim dairy, poultry, or rabbit; Martinez-Ferran et al., 2020). In addition, vitamin D supplementation would also be justified due to the low sun exposure that is usually received during confinement (Nair and Maseeh, 2012). These nutritional guidelines reduced the possibility of suffering from chronic diseases and increased the chance having a strong immune system that ensures a return to optimal normality and being prepared to face a relapse of COVID-19 or any other disease.

### Gut Microbiome Alterations Due to COVID-19 and Its Brain Nexus

The gut microbiome has been defined as the second human brain, due to its bidirectional signaling between the gastrointestinal tract and the brain, being vital for homeostasis maintenance and involved in the production of brain transmitters such as serotonin, dopamine, GABA, norepinephrine, or acetylcholine (Ridaura and Belkaid, 2015).

Current scientific evidence shows that the gut microbiota plays an important role in the development of mental disorders such as depression, anxiety, Alzheimer’s, Parkinson’s, obsessive-compulsive disorder, eating disorders, autism spectrum disorders, multiple sclerosis, and epilepsy (Kelly et al., 2015). In this line, stress has proven to influence the composition and function of gut microbiota (Foster et al., 2017). The high co-morbidity between stress-related psychological symptoms, such as anxiety, with gastrointestinal disorders, including irritable bowel disorder, and inflammatory bowel disorder (Cámara et al., 2009), is strong evidence of this axis (Cryan and O’mahony, 2011). Animal studies on germ-free mice reported altered anxiety-related behavior, supporting the link between microbiota and stress (De Palma et al., 2014), as well as increased hypothalamic-pituitary-adrenal axis (HPA) activity (Luczynski et al., 2016).

Recent studies suggest how psychological stress may also increase the production of hypothalamic and amygdala corticotropin-releasing hormone (CRH), which has been related as a precursor of the HPA axis, thus leading to cortisol production (Vanuytsel et al., 2014). Since CRH has an impact on the production of inflammatory cytokines and tumor necrosis factor-alpha (TNF-α), it is likely to underpin the association of stress and stress-associated medical conditions with the novel SARS-CoV-2, therefore increasing the severity of and fatalities associated with the COVID-19 pandemic. Thus, by such effects on the immune system and the reactions it may trigger, the gut microbiome may modulate the pathophysiology of SARS-CoV-2 (Anderson, 2020). In addition, most of the medical conditions that have been pointed as causing an increased risk of fatality from SARS-CoV-2 are associated with alterations either on gut permeability and dysbiosis, such as obesity, diabetes, cardiovascular disorders, and lung and respiratory airways inflammation (Anderson and Maes, 2020).

In this line, the increased level of pro-inflammatory cytokines presents a direct effect on the downregulation of hormones such as serotonin and melatonin, which are necessary for the maintenance of circadian rhythms (Markus et al., 2018). This situation suggests that stress will make the population more susceptible to the viral infection by an affection of the second brain, the gut microbiome (Ridaura and Belkaid, 2015). COVID-19 is pulmonary, a fact that highlights the importance of gut, since previous authors already found a “gut-lung axis” (Keely et al., 2012), suggesting how respiratory infections are associated with a change in the composition of the gut microbiota (Groves et al., 2020). This exposes a bidirectional relation, in which microbial metabolites and certain endotoxins may impact the lung through blood and when inflammation may happen on the lung, reciprocally affecting the gut microbiota (Dumas et al., 2018). This in turn raises an interesting possibility that novel SARS-Cov2 might also have an impact on the gut microbiota.

## Physiological Sequelae of the COVID-19

Coronavirus Disease 2019 infection in humans can lead to mild symptoms, with a recovery time of 1 to 2 weeks, to more severe cases which may result in death (Adhikari et al., 2020). That being said, the magnitude of symptoms is related to the health status before infection. As such, elderly men and people with underlying cardiovascular and respiratory diseases and cancer (Tan and Aboulhosn, 2020; Wu and McGoogan, 2020), obese individuals (Petrakis et al., 2020), and those with diabetes mellitus (Muniyappa and Gubbi, 2020) are more susceptible to infection. Nevertheless, the relative importance of these health conditions is yet unknown. Infants, children, and young adults (20- to 54-years-old) are also likely to be infected (Bialek et al., 2020; Lu et al., 2020). While cases of cardiomyopathy have been reported in pregnant women (Juusela et al., 2020), this population group is not considered a high-risk group; however, more studies are required (Fang et al., 2020).

The most common symptoms include fever, dry cough, dyspnea, arthralgia, and myalgia (Zhang J.J. et al., 2020). Recently two more symptoms, loss of taste and loss of smell, were identified as markers of mild to moderate infection (Lechien et al., 2020). Less common symptoms include headache, hemoptysis, rhinorrhea, and gastrointestinal symptoms such as abdominal pain, diarrhea, and nausea (Di Gennaro et al., 2020). In more severe cases, lymphopenia is acknowledged (Tan et al., 2020; Figure 2). Moreover, arrhythmias and acute kidney injury have been reported in COVID-19 patients (Fanelli et al., 2020; Guo et al., 2020), as well as the production of blood clots, ultimately causing a pulmonary embolism and thrombosis (Wise, 2020). Finally, regarding older individuals, the potential link and implications of gut microbiota (Dhar and Mohanty, 2020), as well as cachexia and sarcopenia (Morley et al., 2020), in severe cases during the recovery phase are discussed.

The chronic physiological sequelae of this pandemic will not be fully understood for some time. Since no viable vaccine is currently available and coronavirus cases are growing exponentially, continued worldwide research is needed in this challenging period to analyze the potential COVID-19-associated health issues and provide detailed and specific guidance.

## Psychological Sequelae of the COVID-19

A large amount of fear, despair, and death has been spread worldwide. The unknown consequences, symptoms, and condition of the virus, the lack of a vaccine, and the collapse of the economic system which is leading to a worldwide recession, is causing mass fear since we are facing the most serious pandemic of the last 100 years (Thakur and Jain, 2020). The dramatic and exponential increase in infections, which could only be countered by drastic decisions such as confinement and quarantine of the entire population, has forced a radical change in citizen’s lifestyles, which has had psychological impacts on many people (Galea et al., 2020).

**FIGURE 1:**
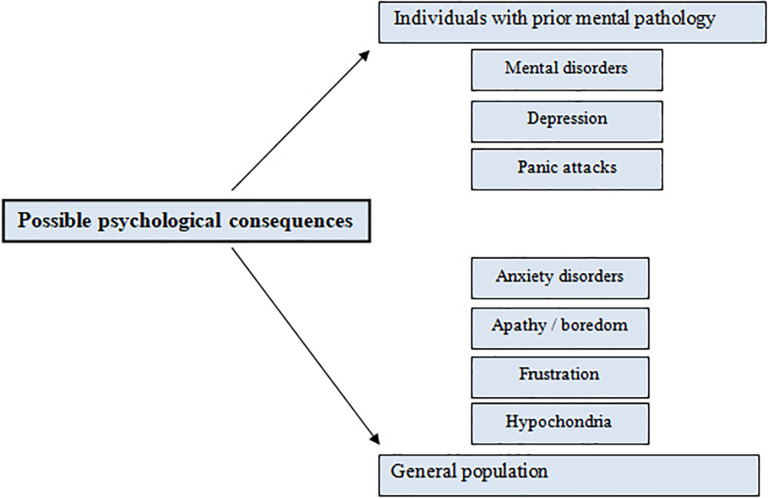
Common and less common physiological symptoms related to the COVID-19 pandemic.

Although it is not yet known exactly what the psychological impact of these drastic measures will be, it is possible to infer some psychological consequences (Figure 1). Firstly, it is important to consider the psychological and emotional state of vulnerable people who, prior to the emergence of the pandemic, had already been diagnosed with some mental pathology (Pfefferbaum and North, 2020). In these cases, it is possible that confinement, social isolation, and the impossibility of continuing with their life routine and habits, may lead subjects with depressive symptoms or other mental disorders to suffer panic attacks due to the sudden stop of activity and the extraordinary situation (Torales et al., 2020). In this line, support is highly necessary, either professional or familiar, through offering therapy or ensuring that they do not worsen over time and that there are not decompensations at a psychopathological level that may lead these patients to address anxiety through disruptive autolytic behaviors (Alradhawi et al., 2020).

Secondly, adolescents are another high-risk group. There is an increase in rates of depression, panic disorder, agoraphobia, and substance use disorders, as well as a decrease in separation anxiety disorder and attention-deficit hyperactivity disorder in the transition from childhood to adolescence (Costello et al., 2011). Major depression and psychological disorders increased with the rise in ownership of smartphones and the consumption of digital media and social networks (Twenge et al., 2019), factors which increased during the pandemic. The confinement made social connections difficult, connections which form the basis for the self-affirmation of adolescents’ personality (Lebel et al., 2020), and the isolation could negatively affect adolescent’s relationships, especially when they did not previously have a good relationship with their relatives (Rosen et al., 2020). In these cases, when the adolescent is in the situation of not being able to correctly face the demands of such an exceptional situation, aggressive behaviors may be found, which may become a risk for people around them and also for themselves. In this sense, different organizations call for attention to the fact that, during the months of confinement, the percentage of suicide attempts by this group has increased from 1.9% to 8.3% (Assari and Habibzadeh, 2020).

**FIGURE 2:**
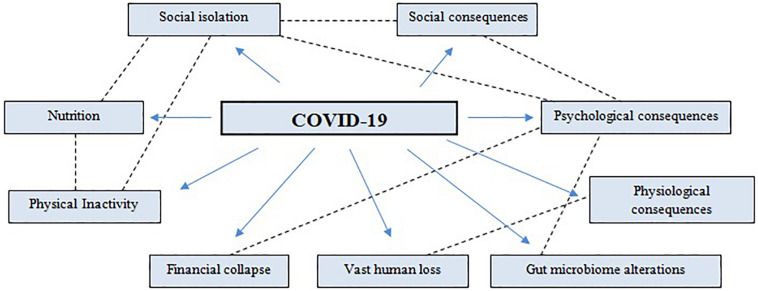
Possible psychological consequences related to the COVID-19 pandemic.

As for the general population, many who previously had not suffered any kind of pathological symptomatology before the pandemic will find themselves developing symptoms. It was estimated that a high percentage of the general population could present psychological sequelae such as anxiety disorders, apathy, boredom, loneliness, and frustration due to the confinement, hypochondria due to fear of contagion, insomnia or sleep disorders, difficulties in the proper functioning of concentration, memory or attention, and may even present symptoms associated with post-traumatic stress disorder (Jia et al., 2020; Figure 2). These consequences will silently appear, as individuals return to the so-called “new normality,” then, researchers will start talking again about a new pandemic, the psychological scar of COVID-19 in our society.

A correct understanding of pleasure and pain, happiness, and virtue gained from Stoic philosophical thought could be applied to the current pandemic and sociological context and be used to cope with the situation.

Today’s society skids, since it has been seduced by the wrong lines of thought (hedonism), instead of following a stoic path. The current western man has let hedonism win the ideological and consequently today we pursue pleasure, forgetting about the consequences that it has. We have undoubtedly created a world that seeks stimulation for stimulation and pleasure for pleasure. Consequently, when all of this is taken from us and a pandemic strikes, our lives are emptier than ever, since no one prepared us for misfortune.

We despair the lack of instant, free, superficial, and fleeting pleasure that defines today’s hedonistic society. All this, because we chose to follow a philosophical line of thought which does not prepare us for difficult times since, from a very young age, we have been taught that the immediate satisfaction of the drives is the right way to go. We only paid attention to Hedoné and not to Areté.

Stoicism teaches us that situations which we are not capable of controlling should not matter to us, since only we are the owners of our thoughts and emotions. The objective must be marked to those elements which we can change. In this sense, the pandemic is irrelevant; it is how we face it that really matters.

Seneca pursued poverty voluntarily in order to appreciate wealth when it was available to him, so in this order…Allow yourself to enjoy confinement to appreciate freedom, that freedom that we take for granted. Let us not be afraid of the loneliness that we now live, since it will make us value future company. Do not fear the inaction that the pandemic brought us because it will show us that the work that we once believed to be a torment was not really one. Enjoy missing family and friends, as this will have taught us how much we love them. Appreciate how cinemas, shops, theaters, and bars are closed so that in this way we will understand what place all this occupied in our lives, in order to establish future priorities. Let’s avoid suffering from inefficiencies since when these comforts are missing some other day, everything will matter and affect us much less. See the obstacles as opportunities. Being locked up offers limitless opportunities for study, to pick up old friendships, to discover new skills, or simply to spend time in solitude in the guise of what you can offer yourself.

Thus, confinement does not have a positive or negative charge, since the only thing that matters for the Stoic is the reaction to events. A stoic does not judge facts or events, but sees how confinement occurs and accepts it as it is, tries to use all their resources to change things if necessary, never becoming that fly that hits the glass, over and over again. There are millions of factors that affect the possibility of our wishes being fulfilled, but a stoic will always have the certainty of controlling that part of things that only depends on him. Let us never hope that the world is as we wish it to be, if not as it really is, in this way we will have a peaceful life, that with ourselves, is enough, since the only way of getting out of the mud, is leaving by our own foot.

## Conclusion

**FIGURE 3:**
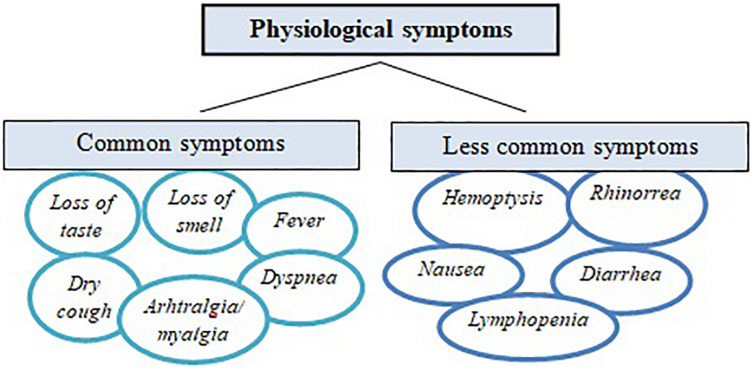
Social and psychophysiological consequences of the COVID-19 pandemic.

Depression, anxiety, panic attacks, somatic symptoms, and PTSD symptoms, delirium, psychosis, and even suicidality may be experienced according to the psychological and psychosocial stressors/triggers. The fear and normalization process of the new reality creates an extremely stressful and novel context for the population. Confinement and restriction measures translates to a sudden stop of citizens’ normal life. thus the person quickly begins to present symptoms associated with an anxiety state produced by social isolation, lack of mental hygiene habits, and repeated exposure to negative news and information. Feelings of sadness, apathy, fear, uncertainty, frustration, lack of impulse control, anxiety, alterations of circadian cycles, insomnia, hypervigilance, or difficulties in concentrating may appear. These symptoms would be aggravated if the person presents mental pathologies previous to the pandemic. The experience of COVID symptomatology may trigger shame, social stigma, and even the feeling of guilt. Moreover, the presence of some symptoms, such as headaches, migraines, or irritating cough, can be mistaken by the subject as COVID symptoms, despite not having the virus; this and the impossibility of performing individual PCR or rapid tests to know if he is infected or not, adds to an even greater state of uncertainty, exponentially increasing psychological distress. Another potential trigger is the grieving process, which is a completely normal and expected reaction which reflects a unique convergence of responses, from affective, behavioral, physical, and cognitive ones. However, unexpected and sudden deaths and the inability to attend the last hours of life and to provide a decent burial, lead to the impossibility of gaining closure to the emotional link, leading to an ambiguous loss, thus stimulating feelings of resentment. Since the world economy is entering into recession, the reality after this situation is showing a catastrophic effect on economies and a vast loss of employment, strongly affecting the mental health of workers, which may translate into depression, anxiety, psychological distress, and a decreased life satisfaction. The social isolation measures imposed by governments across the world caused an abrupt decline in physical activity, as recreation facilities, athletic centers, gyms, public parks, playgrounds, and schools were forced to close. However, physical activity is more than encouraged, since it has been proven to enhance the immune system, prevent respiratory infections, and consequently, prevent new COVID-19 incidences. An excess of energy in a situation of physical inactivity can increase metabolic disorders that in turn increases the risk of multiple chronic diseases; thus appropriate nutrition is essential and it is recommended to have a balanced and healthy diet with a restricted calorie intake, avoiding an over-intake. Finally, gut microbiome may be a potential key factor (Figure 3), since the gut microbiota plays an important role in the development of mental disorders such as depression and anxiety (strongly present as a consequence of the pandemic) and the bidirectional relation, in where microbial metabolites and certain endotoxins may impact the lung through blood and when inflammation may happen on the lung, reciprocally affects the gut microbiota, raises an interesting focus point.

## Author Contributions

VC-S and JT-A: conceptualization. JM-A: methodology. AD: writing—original draft preparation all, writing—review and editing, all. VC-S and JT-A: supervision. All authors contributed to the article and approved the submitted version.

## Conflict of Interest

The authors declare that the research was conducted in the absence of any commercial or financial relationships that could be construed as a potential conflict of interest.
